# Supplementary material for: Mechanical Models of Pattern and Form in Biological Tissues: The Role of Stress–Strain Constitutive Equations
Source: Bull Math Biol. 2021 May 26;83(7):80. doi: 10.1007/s11538-021-00912-5 (PMC8154836; doi:10.1007/s11538-021-00912-5)
Supplement: Supplementary file 7 — Supplementary material 7 (pdf 252 KB) [file 11538_2021_912_MOESM7_ESM.pdf]

Mechanical models of pattern and form in biological tissues: the role of stress-strain constitutive equations* Supplementary Information: Numerical Schemes

Chiara Villa Mark A. J. Chaplain† Alf Gerisch Tommaso Lorenzi

In this document (supplementary information) we report on details of the numerical schemes employed to obtain the numerical results presented in the above named publication (main publication). For details of the equations referred to in this document, please see the main publication. Numerical solutions are computed using MATLAB, and the files containing the code corresponding to the schemes presented below can be found on GitLab (`https://git-ce.rwth-aachen.de/alf.gerisch/VillaEtAl2021BullMathBiol`).

Numerical schemes for the system of PDEs (10), (11) and (16)

Numerical solutions for the system of implicit, time-dependent and spatially one-dimensional PDEs (10), (11) and (16) are obtained exploiting the Method of Lines. We make use of a uniform discretisation of the spatial domain $[l, L]$ consisting of $K + 1$ grid points, or grid cell centres, while, at first, leaving the time variable continuous. We denote the spatial grid width by Δx. The normalised cell density $n(t, x)$, the normalised ECM density $\rho(t, x)$ and the displacement of a material point of the cell-ECM system $u(t, x)$ are approximated as

$$n(t, x_i) \approx N_i(t)\,, \quad \rho(t, x_i) \approx P_i(t)\,, \quad u(t, x_i) \approx U_i(t) \quad \text{for } i = 0, \dots, K\,.$$

Thanks to the periodic boundary conditions we have

$$N_0(t) = N_K(t)\,, \quad P_0(t) = P_K(t) \quad \text{and} \quad U_0(t) = U_K(t)\,,$$

and consequently have $3 \times K$ time-continuous approximations to determine. We collect them in the vectors $N(t)$, $P(t)$, $U(t)$ and denote their time-derivatives by $N'(t)$, $P'(t)$, $U'(t)$. The discretization of the spatial derivatives in the PDE system will then result in an implicit system of $3 \times K$ ODEs for the variables $N(t)$, $P(t)$, $U(t)$ and their time-derivatives of the following form

$$F(N, P, U, N', P', U') = \begin{bmatrix} f_n(N, P, N', U') \\ f_\rho(P, P', U') \\ f_u(N, P, U, N', P', U') \end{bmatrix} = 0\,. \tag{S.1}$$

In this system $f_n(N, P, N', U') = 0$, $f_\rho(P, P', U') = 0$ and $f_u(N, P, U, N', P', U') = 0$ are each systems of K ODEs obtained, respectively, from PDEs (10), (11) and (16), using second-order central finite difference approximations for the spatial derivatives and the first-order upwind scheme for the advection terms, as detailed below for each equation.

In order to solve system (S.1), we make use of the MATLAB solver `ode15i`, which uses a variable-order (orders 1 to 5) backward difference formula (BDF) method in a form suitable to an implicit system

*This paper is published in *Bulletin of Mathematical Biology*.

†Corresponding author affiliation and email: Mark A. J. Chaplain, School of Mathematics and Statistics, University of St Andrews, St Andrews KY16 9SS (UK), majc@st-andrews.ac.uk

of ODEs. Initial conditions $N(0)$, $P(0)$ and $U(0)$ are given by the appropriate equivalent of initial conditions (28), and we make use of the MATLAB function `decic` to obtain consistent initial conditions $N'(0)$, $P'(0)$ and $U'(0)$ such that (S.1) is satisfied at initial time $t = 0$.

Useful matrices. In order to apply the first-order upwind scheme we need to compute variables and derivatives at the grid cell interfaces, *i.e.* half-way between grid points, in addition to those at the grid cell centres. We here clarify the notation adopted throughout the rest of this document.
The $K\times K$ matrices $\mathbf{M}_x$ and $\mathbf{M}_{xx}$ are used to approximate, using second-order finite differences, the first-order and the second-order derivatives in space, respectively, of a periodic grid function at the grid cell centres and are therefore given by

$$\mathbf{M}_x := \frac{1}{2\Delta x}\begin{bmatrix} 0 & 1 & & & -1 \\ -1 & 0 & 1 & & \\ & \ddots & \ddots & \ddots & \\ & & -1 & 0 & 1 \\ 1 & & & -1 & 0 \end{bmatrix} \quad \text{and} \quad \mathbf{M}_{xx} := \frac{1}{\Delta x^2}\begin{bmatrix} -2 & 1 & & & 1 \\ 1 & -2 & 1 & & \\ & \ddots & \ddots & \ddots & \\ & & 1 & -2 & 1 \\ 1 & & & 1 & -2 \end{bmatrix}. \tag{S.2}$$

We make use of the notation $\overrightarrow{\cdot}$ to indicate a shift from the grid cell centres to the (right) grid cell interfaces. In particular to approximate the value of a periodic grid function at these grid cell interfaces we multiply it by the $K\times K$ matrix

$$\overrightarrow{\mathbf{M}} := \frac{1}{2}\begin{bmatrix} 1 & 1 & & & \\ & 1 & 1 & & \\ & & \ddots & \ddots & \\ & & & 1 & 1 \\ 1 & & & & 1 \end{bmatrix}. \tag{S.3}$$

In addition, the $K\times K$ matrices $\overrightarrow{\mathbf{M}}_x$ and $\overleftarrow{\mathbf{M}}_x$ are used to approximate the first-order derivatives in space of a periodic grid function at the (right) grid cell interfaces, when the grid function is given in the grid cell centres, and at the grid cell centres, when the grid function is given in the (right) grid cell interfaces, respectively. These are given by

$$\overrightarrow{\mathbf{M}}_x := \frac{1}{\Delta x}\begin{bmatrix} -1 & 1 & & & \\ & -1 & 1 & & \\ & & \ddots & \ddots & \\ & & & -1 & 1 \\ 1 & & & & -1 \end{bmatrix} \quad \text{and} \quad \overleftarrow{\mathbf{M}}_x := \frac{1}{\Delta x}\begin{bmatrix} 1 & & & & -1 \\ -1 & 1 & & & \\ & \ddots & \ddots & & \\ & & -1 & 1 & \\ & & & -1 & 1 \end{bmatrix}. \tag{S.4}$$

Note that, even though these two matrices are multiplied by $1/\Delta x$, they still stem from second-order finite difference approximations, calculated on a staggered grid shifted by half the grid cell width.
Convention: In the formulas which follow below, we use the convention that any product of a matrix from above with a vector of length K is a matrix-vector product but any operation between two vectors, in particular multiplication, division, or exponentiation, are understood element-wise.

Numerical scheme for the balance equation (10). We rewrite the balance equation (10) as

$$\partial_t n - D\partial^2_{xx} n + \partial_x(\phi\, n) - rn(1-n) = 0 \quad \text{with} \quad \phi = \alpha\,\partial_x\rho + \partial_t u\,,$$

which, upon spatial discretisation, leads to the following system of K ODEs

$$f_n(N, P, N', U') = N' - D\,\mathbf{M}_{xx}\,N + \mathrm{A}\big(\overrightarrow{\Phi}, N\big) - rN(1-N) = 0 \tag{S.5}$$

with $\overrightarrow{\Phi}$ indicating the advective velocity computed at the grid cell interfaces, that is

$$\overrightarrow{\Phi} = \alpha\, \overrightarrow{\mathbf{M}}_x\, P + \overrightarrow{\mathbf{M}}\, U' , \tag{S.6}$$

and the matrices $\mathbf{M}_{xx}$, $\overrightarrow{\mathbf{M}}$ and $\overrightarrow{\mathbf{M}}_x$ are defined in (S.2), (S.3) and (S.4), respectively. The function $\mathrm{A}\big(\overrightarrow{\Phi}, N\big)$ computes the contribution of advection, given advective velocity and advected quantity as inputs, at the grid cell centres as

$$\mathrm{A}\big(\overrightarrow{\Phi}, N\big) := \overleftarrow{\mathbf{M}}_x\, \overrightarrow{\mathscr{F}}\big(\overrightarrow{\Phi}, N\big) \tag{S.7}$$

where the matrix $\overleftarrow{\mathbf{M}}_x$ is defined in (S.4) and the advective flux $\overrightarrow{\mathscr{F}}$ at the grid cell interfaces is computed using first-order upwinding, *i.e.*

$$\Big[\overrightarrow{\mathscr{F}}\big(\overrightarrow{\Phi}, N\big)\Big]_i := \begin{cases} \big(\overrightarrow{\Phi}_i\big)^+ N_i + \big(\overrightarrow{\Phi}_i\big)^- N_{i+1} & \text{for } i = 1, ..., K-1 \\ \big(\overrightarrow{\Phi}_K\big)^+ N_K + \big(\overrightarrow{\Phi}_K\big)^- N_1 & \text{for } i = K \end{cases} \tag{S.8}$$

with $(\cdot)^+$ and $(\cdot)^-$ being the positive and negative parts of the input variable, *i.e.*

$$(\Phi)^+ := \max(0, \Phi) \qquad \text{and} \qquad (\Phi)^- := \min(0, \Phi)\ . \tag{S.9}$$

Numerical scheme for the transport equation (11). We rewrite the transport equation (11) as

$$\partial_t \rho\, +\, \partial_x(\partial_t u\, \rho)\, =\, 0$$

which, upon spatial discretisation, leads to the following system of K ODEs

$$f_\rho(P, P', U') = P' + \mathrm{A}\big(\overrightarrow{\mathbf{M}}\, U', P\big)\, = 0\ , \tag{S.10}$$

where the function $\mathrm{A}\big(\overrightarrow{\mathbf{M}}\, U', P\big)$ is defined in (S.7), together with definitions (S.8) and (S.9), with advection velocity given by U' calculated at the cell interfaces using $\overrightarrow{\mathbf{M}}$ defined in (S.3).

Numerical scheme for the force-balance equation (16). We solve the system of PDEs (10), (11) and (16) for the Kelvin-Voigt (3) and the Maxwell (4) models. In these cases we have $b_2 = a_2 = 0$, and the force-balance equation (16) reads as

$$b_1\, \partial^3_{xxt} u + b_0\, \partial^2_{xx} u\, -\, a_1 s\, \partial_t(\rho u)\, -\, a_0\, s\rho u\, +\, \partial_x\big(a_1\, \partial_t \sigma_c + a_0\, \sigma_c\big) = 0 \quad \text{with} \quad \sigma_c = \tau\, \frac{n}{1 + \lambda\, n^2}\big(\rho + \beta\, \partial^2_{xx}\rho\big)\, .$$

Upon spatial discretisation, this leads to the following system of K ODEs

$$f_u(N, P, U, N', P', U') = b_1 \mathbf{M}_{xx} U' + b_0 \mathbf{M}_{xx} U - a_1 s (PU)' - a_0 s P U + \mathbf{M}_x \mathrm{T}_1(N, P, N', P') = 0 \tag{S.11}$$

with

$$\mathrm{T}_1(N, P, N', P') = \ \tau \big[\, a_1\, \Lambda_2(N) N' \mathbf{M}_{T1} P + a_1\, \Lambda_1(N) \mathbf{M}_{T1} P' + a_0\, \Lambda_1(N) \mathbf{M}_{T1} P\, \big]\, , \tag{S.12}$$

where the functions Λ_1 and Λ_2 are defined as

$$\Lambda_1(N) := \frac{N}{1 + \lambda\, N^2} \qquad \text{and its derivative} \qquad \Lambda_2(N) := \frac{1 - \lambda\, N^2}{(1 + \lambda\, N^2)^2}\, , \tag{S.13}$$

while the $K{\times}K$ matrix $\mathbf{M}_{T1}$ is given by

$$\mathbf{M}_{T1} := \mathbf{I} + \beta\, \mathbf{M}_{xx}\, , \tag{S.14}$$

where $\mathbf{I}$ is the $K{\times}K$ identity matrix and $\mathbf{M}_x$ is defined in (S.2).
This scheme is valid as long as $b_2 = a_2 = 0$ and can therefore also be applied when considering the linear elastic model (1), the linear viscous model (2), and the SLS model (5). On the other hand, in the case where $b_2 \neq 0$ (*i.e.* when the Jeffrey model (6) is considered) the above numerical scheme cannot be directly employed due to the presence of a second-order derivative in t. We could still, however, take a similar approach and make use of the `ode15i` solver by introducing extra variables for the first-order derivatives in t of n and ρ, thus formally reducing the PDE (16) to first-order in time, at the cost of increasing the number of equations in the Method of Lines ODE system.

Numerical scheme for the system of PDEs (29)

Similarly as done for the spatially one-dimensional model, numerical solutions for the system of implicit, time-dependent and spatially two-dimensional PDEs (29), together with (30)-(32), are obtained exploiting the Method of Lines. We make use of a uniform discretisation of the square spatial domain $[l, L] \times [l, L]$ consisting of $(K+1)\times(K+1)$ grid points, while leaving the time variable continuous. The spatial grid width, in both spatial directions, is denoted by Δx again. The normalised cell density $n(t, x_1, x_2)$, the normalised ECM density $\rho(t, x_1, x_2)$ and the displacement of a material point of the cell-ECM system $\boldsymbol{u}(t, x_1, x_2) = \big(u_1(t, x_1, x_2), u_2(t, x_1, x_2)\big)^{\mathsf{T}}$ are approximated as

$$n(t, x_i, x_j) \approx N_{i,j}(t)\,, \quad \rho(t, x_i, x_j) \approx P_{i,j}(t) \quad \text{for } i, j = 0, \ldots, K\,,$$

$$u_1(t, x_i, x_j) \approx (U_1)_{i,j}(t)\,, \quad u_2(t, x_i, x_j) \approx (U_2)_{i,j}(t) \quad \text{for } i, j = 0, \ldots, K\,.$$

Thanks to the periodic boundary conditions, we can drop the index values $i = 0$ and $j = 0$ and consequently have $4 \times K^2$ time-continuous approximations to determine. We collect them in the matrices $N(t)$, $P(t)$, $U_1(t)$, $U_2(t)$ and denote their time-derivatives by $N'(t)$, $P'(t)$, $U_1'(t)$, $U_2'(t)$. The discretization of the spatial derivatives in the PDE system will then result in an implicit system of $4 \times K^2$ ODEs for the variables $N(t)$, $P(t)$, $U_1(t)$, $U_2(t)$ and their time-derivatives of the following form

$$F(N, P, U_1, U_2, N', P', U_1', U_2') = \begin{bmatrix} f_n(N, P, N', U_1', U_2') \\ f_\rho(P, P', U_1', U_2') \\ f_{u_1}(N, P, U_1, U_2, N', P', U_1', U_2') \\ f_{u_2}(N, P, U_1, U_2, N', P', U_1', U_2') \end{bmatrix} = 0\,. \tag{S.15}$$

In this system $f_n(N, P, N', U_1', U_2') = 0$, $f_\rho(N, P, N', U_1', U_2') = 0$, $f_{u_1}(N, P, U_1, U_2, N', P', U_1', U_2') = 0$ and $f_{u_2}(N, P, U_1, U_2, N', P', U_1', U_2') = 0$ are each systems of K^2 ODEs obtained from the system of PDEs (29), using second-order central finite difference approximations for the spatial derivatives and the first-order upwind scheme for the advection terms, as detailed below for each equation.

In order to solve system (S.15), we make, similarly to the spatially one-dimensional case, use of the Matlab solver `ode15i`. Initial conditions $N(0)$, $P(0)$, $U_1(0)$ and $U_2(0)$ are given by the appropriate equivalent of initial conditions (36), and we make use of the Matlab function `decic` to obtain consistent initial conditions $N'(0)$, $P'(0)$, $U_1'(0)$ and $U_2'(0)$ such that (S.15) is satisfied at initial time $t = 0$.

Useful functions In order to solve the system (S.15) we need to compute variables and derivatives at the grid cell centers and interfaces, both in the x_1- and the x_2-direction. We here introduce the functions that will be used in the rest of this document to compute the aforementioned quantities in the different directions. These rely on the fact that the matrices (S.2)-(S.4) act on column vectors and therefore, when applied to an $K\times K$ argument matrix, they will act on each column of that, which in our framework corresponds to computing the quantity of interest in the x_1-direction. In order to compute the same quantities in the x_2-direction, we need the operating matrix to act on each row of the argument matrix of interest, which can be achieved by matrix transposition of the argument matrix before and of the product matrix after matrix multiplication. Hence the functions $\mathbf{M}_{x1}(N)$ and $\mathbf{M}_{x2}(N)$ are used to approximate the first-order derivative of the variable of interest, say N, at the grid cell centres in the x_1- and x_2-directions respectively, and are defined as

$$\mathbf{M}_{x1}(N) := \mathbf{M}_x\, N\,, \qquad \text{and} \qquad \mathbf{M}_{x2}(N) := \big[\mathbf{M}_x\, N^{\mathsf{T}}\big]^{\mathsf{T}}\,, \tag{S.16}$$

where the matrix $\mathbf{M}_x$ is defined in (S.2). Similarly, the functions $\mathbf{M}_{xx1}(N)$ and $\mathbf{M}_{xx2}(N)$ are used to approximate the second-order derivative of the variable of interest at the grid cell centres in the x_1- and x_2-directions, respectively, and are defined as

$$\mathbf{M}_{xx1}(N) := \mathbf{M}_{xx}\, N\,, \qquad \text{and} \qquad \mathbf{M}_{xx2}(N) := \big[\mathbf{M}_{xx}\, N^{\mathsf{T}}\big]^{\mathsf{T}}\,, \tag{S.17}$$

where the matrix $\mathbf{M}_{xx}$ is defined in (S.2). Then the function $\mathbf{M}_{x1x2}(N)$ is used to approximate the second-order mixed derivative in space at the grid cell centres and is defined as

$$\mathbf{M}_{x1x2}(N) := \mathbf{M}_{x2}\big(\mathbf{M}_{x1}(N)\big) = \big[\mathbf{M}_x \left(\mathbf{M}_x N\right)^{\intercal}\big]^{\intercal} . \tag{S.18}$$

In order to approximate the value of a variable in the centres of the (right or upper) grid cell interfaces in the x_1- and x_2-direction, we make use of the functions $\overrightarrow{\mathbf{M}}_1$ and $\overrightarrow{\mathbf{M}}_2$, respectively, which are defined as

$$\overrightarrow{\mathbf{M}}_1(N) := \overrightarrow{\mathbf{M}}\, N , \qquad \text{and} \qquad \overrightarrow{\mathbf{M}}_2(N) := \big[\overrightarrow{\mathbf{M}}\, N^{\intercal}\big]^{\intercal} , \tag{S.19}$$

with the matrix $\overrightarrow{\mathbf{M}}$ defined in (S.3). In a similar fashion we define the functions $\mathrm{A}_1\big(\overrightarrow{v_1}, N\big)$ and $\mathrm{A}_2\big(\overrightarrow{v_2}, N\big)$ which approximate the contribution of advection in the x_1- and x_2-direction, respectively, given as input the advective velocity at the grid cell interfaces in the direction of interest – say v_1 and v_2 are, respectively, the first and second components of the advective velocity – and the advected quantity. These are given by

$$\mathrm{A}_1\big(\overrightarrow{v_1}, N\big) := \mathrm{A}\big(\overrightarrow{v_1}, N\big) \qquad \text{and} \qquad \mathrm{A}_2\big(\overrightarrow{v_2}, N\big) := \big[\mathrm{A}\big(\overrightarrow{v_2}^{\intercal}, N^{\intercal}\big)\big]^{\intercal} , \tag{S.20}$$

with the function $\mathrm{A}\big(\overrightarrow{v_1}, N\big)$ given by (S.7) together with definitions (S.8) and (S.9).
Convention: With the definitions above, we have hidden all applications of the matrices from the spatially one-dimensional case in newly defined functions. Consequently, in the formulas which follow below, we use the convention that any further operation between matrices, in particular multiplication, division, or exponentiation, are understood element-wise.

Numerical scheme for the balance equation (29)$_1$. We rewrite the balance equation $(29)_1$ as

$$\partial_t n - D\big[\partial^2_{x_1x_1} n + \partial^2_{x_2x_2} n\big] + \partial_{x_1}(\phi_1\, n) + \partial_{x_2}(\phi_2\, n) - rn(1-n) = 0 \quad \text{with} \quad \phi_i = \alpha\, \partial_{x_i}\rho + \partial_t u_i \;\; i = 1, 2 ,$$

which, upon spatial discretisation, leads to the following system of K^2 ODEs

$$f_n(N, P, N', U_1', U_1') = N' - D\big[\mathbf{M}_{xx1}(N) + \mathbf{M}_{xx2}(N)\big] + \mathrm{A}_1\big(\overrightarrow{\Phi}_1, N\big) + \mathrm{A}_2\big(\overrightarrow{\Phi}_2, N\big) - rN(1-N) = 0 \tag{S.21}$$

with the functions $\mathbf{M}_{xx1}(\cdot)$ and $\mathbf{M}_{xx2}(\cdot)$ defined in (S.17), and the components of the advective velocity at the grid cell interfaces given by

$$\overrightarrow{\Phi}_1 = \alpha\, \overrightarrow{\mathbf{M}}_{x1}(P) + \overrightarrow{\mathbf{M}}_1(U_1') \quad \text{and} \quad \overrightarrow{\Phi}_2 = \alpha\, \overrightarrow{\mathbf{M}}_{x2}(P) + \overrightarrow{\mathbf{M}}_2(U_2') , \tag{S.22}$$

where functions $\overrightarrow{\mathbf{M}}_{x1}(\cdot)$ and $\overrightarrow{\mathbf{M}}_{x2}(\cdot)$ are defined in (S.16), $\overrightarrow{\mathbf{M}}_1(\cdot)$ and $\overrightarrow{\mathbf{M}}_2(\cdot)$ are defined in (S.19), and functions $\mathrm{A}_1(\cdot,\cdot)$ and $\mathrm{A}_2(\cdot,\cdot)$ are defined in (S.20).

Numerical scheme for the transport equation (29)$_2$. We rewrite the transport equation $(29)_2$ as

$$\partial_t\rho + \partial_{x_1}(\partial_t u_1\, \rho) + \partial_{x_2}(\partial_t u_2\, \rho) = 0$$

which, upon spatial discretisation, leads to the following system of K^2 ODEs

$$f_\rho(P, P', U_1', U_2') = P' + \mathrm{A}_1\big(\overrightarrow{\mathbf{M}}_1(U_1'), P\big) + \mathrm{A}_2\big(\overrightarrow{\mathbf{M}}_2(U_2'), P\big) = 0 , \tag{S.23}$$

where the functions $\mathrm{A}_1(\cdot,\cdot)$ and $\mathrm{A}_2(\cdot,\cdot)$ are defined in (S.20) and functions $\overrightarrow{\mathbf{M}}_1(\cdot)$ and $\overrightarrow{\mathbf{M}}_2(\cdot)$ are defined in (S.19).

Numerical scheme for the force balance equation $(29)_3$. We rewrite the first component of the force balance equation $(29)_3$, complemented with (30)-(32), as

$$\begin{aligned} & b_1\Big(\partial^2_{x_1x_1}\partial_t u_1 + \frac{1}{2}\big[\partial^2_{x_2x_2}\partial_t u_1 + \partial^2_{x_1x_2}\partial_t u_2\big]\Big) + b_0\Big(\partial^2_{x_1x_1} u_1 + \frac{1}{2}\big[\partial^2_{x_2x_2} u_1 + \partial^2_{x_1x_2} u_2\big]\Big) + \\ & c_1\big(\partial^2_{x_1x_1}\partial_t u_1 + \partial^2_{x_1x_2}\partial_t u_2\big) + c_0\big(\partial^2_{x_1x_1} u_1 + \partial^2_{x_1x_2} u_2\big) + \\ & \partial_{x_1}[a_1\partial_t\sigma_c + a_0\sigma_c] - a_1 s(u_1\partial_t\rho + \rho\partial_t u_1) - a_0 s\rho u_1 = 0 \,, \end{aligned} \tag{S.24}$$

and, similarly, we rewrite the second component as

$$\begin{aligned} & b_1\Big(\partial^2_{x_2x_2}\partial_t u_2 + \frac{1}{2}\big[\partial^2_{x_1x_2}\partial_t u_1 + \partial^2_{x_1x_1}\partial_t u_2\big]\Big) + b_0\Big(\partial^2_{x_2x_2} u_2 + \frac{1}{2}\big[\partial^2_{x_1x_2} u_1 + \partial^2_{x_1x_1} u_2\big]\Big) + \\ & c_1\big(\partial^2_{x_2x_2}\partial_t u_2 + \partial^2_{x_1x_2}\partial_t u_1\big) + c_0\big(\partial^2_{x_2x_2} u_2 + \partial^2_{x_1x_2} u_1\big) + \\ & \partial_{x_2}[a_1\partial_t\sigma_c + a_0\sigma_c] - a_1 s(u_2\partial_t\rho + \rho\partial_t u_2) - a_0 s\rho u_2 = 0 \,, \end{aligned} \tag{S.25}$$

where σ_c is defined by

$$\sigma_c = \tau\frac{n}{1+\lambda n^2}\big(\rho + \beta\partial^2_{x_1x_1}\rho + \beta\partial^2_{x_2x_2}\rho\big) \,. \tag{S.26}$$

Upon spatial discretisation, these lead to the following systems of K^2 ODEs

$$\begin{aligned} f_{u1}(N,P,U,N',P',U') = {} & b_1\big(\mathbf{M}_{xx1}(U_1') + \frac{1}{2}\big(\mathbf{M}_{xx2}(U_1') + \mathbf{M}_{x1x2}(U_2')\big)\big) + \\ & b_0\big(\mathbf{M}_{xx1}(U_1) + \frac{1}{2}\big(\mathbf{M}_{xx2}(U_1) + \mathbf{M}_{x1x2}(U_2)\big)\big) + \\ & c_1\big(\mathbf{M}_{xx1}(U_1') + \mathbf{M}_{x1x2}(U_2')\big) + c_0\big(\mathbf{M}_{xx1}(U_1) + \mathbf{M}_{x1x2}(U_2)\big) + \\ & \mathbf{M}_{x1}\big(\mathrm{T}_2(N,P,N',P')\big) - a_1 s(PU_1' + P'U_1) - a_0 sPU_1 \;=\; 0 \end{aligned} \tag{S.27}$$

and

$$\begin{aligned} f_{u2}(N,P,U,N',P',U') = {} & b_1\big(\mathbf{M}_{xx2}(U_2') + \frac{1}{2}\big(\mathbf{M}_{x1x2}(U_1') + \mathbf{M}_{xx1}(U_2')\big)\big) + \\ & b_0\big(\mathbf{M}_{xx2}(U_2) + \frac{1}{2}\big(\mathbf{M}_{x1x2}(U_1) + \mathbf{M}_{xx1}(U_2)\big)\big) + \\ & c_1\big(\mathbf{M}_{xx2}(U_2') + \mathbf{M}_{x1x2}(U_1')\big) + c_0\big(\mathbf{M}_{xx2}(U_2) + \mathbf{M}_{x1x2}(U_1)\big) + \\ & \mathbf{M}_{x2}\big(\mathrm{T}_2(N,P,N',P')\big) - a_1 s(PU_2' + P'U_2) - a_0 sPU_2 \;=\; 0 \,. \end{aligned} \tag{S.28}$$

Here

$$\mathrm{T}_2(N,P,N',P') = \; \tau\big[\, a_1\,\Lambda_2(N)N'\,\mathbf{M}_{T2}(P) + a_1\,\Lambda_1(N)\,\mathbf{M}_{T2}(P') + a_0\,\Lambda_1(N)\,\mathbf{M}_{T2}(P)\,\big] \,, \tag{S.29}$$

where the functions Λ_1 and Λ_2 are defined as in (S.13), while the function $\mathbf{M}_{T2}(P)$ is given by

$$\mathbf{M}_{T2}(P) := P + \beta\big[\,\mathbf{M}_{xx1}(P) + \mathbf{M}_{xx2}(P)\,\big] \,, \tag{S.30}$$

where the functions $\mathbf{M}_{xx1}(\cdot)$ and $\mathbf{M}_{xx2}(\cdot)$ are defined in (S.17).

Remark: The MATLAB solver `ode15i` allows for the specification of the sparsity pattern of Jacobian matrices. In particular in the spatially two-dimensional simulations this leads, in comparison to not specifying these patterns, to substantial savings in required CPU time. For details on these patterns we refer to the available MATLAB implementation for the numerical solution of the PDE systems.
